# Impact of demineralization, brushing, and remineralization sequences on dentin mineral retention and surface properties

**DOI:** 10.1007/s10266-025-01070-z

**Published:** 2025-02-26

**Authors:** Soyeon Kim, Sri Larnani, Ji Eun Son, Napas Lappanakokiat, Van Mai Truong, Bo-Hyoung Jin, Young-Seok Park

**Affiliations:** 1 Department of Oral Anatomy and Dental Research Institute, School of Dentistry, Seoul National University, Seoul, Republic of Korea 03080; 2 Department of Integrated Dentistry, School of Dentistry, Seoul National University, Seoul, Republic of Korea 03080; 3 Faculty of Odonto-Stomatology, University of Health Sciences, Vietnam National University, Ho Chi Minh City, Vietnam 700000; 4 Department of Preventative & Public Health Dentistry and Dental Research Institute, School of Dentistry, Seoul National University, Seoul, Republic of Korea 03080; 5 Center for Future Dentistry, School of Dentistry, Seoul National University, Seoul, Republic of Korea 03080

**Keywords:** Dentin, Remineralization, Demineralization, X-ray fluorescence spectroscopy

## Abstract

This study investigates the effects of different treatment sequences involving demineralization, brushing, and remineralization on the calcium and phosphorus (Ca/P) content, surface roughness, and microhardness of dentin specimens. Bovine dentin samples were subjected to the following five treatment conditions: control, demineralization, demineralization followed by remineralization, demineralization followed by brushing then remineralization, and demineralization followed by remineralization then brushing. X-ray fluorescence spectroscopy was then utilized to assess the elementary composition changes, while scanning electron microscopy provided microstructural analyses. Surface roughness and microhardness were also quantified to assess the physical changes in dentin. The control group retained significantly higher Ca/P content compared with all treated groups, indicating that demineralization, regardless of subsequent treatment, leads to a substantial loss of hydroxyapatite. Among the treated groups, those that underwent remineralization immediately after demineralization manifested higher Ca/P retention compared with those that included brushing before remineralization. Additionally, microhardness measurements indicated that post-demineralization brushing negatively affected dentin’s microhardness. The sequence of demineralization, brushing, and remineralization treatments significantly affects Ca/P retention in dentin, along with its surface roughness and microhardness. Pre-remineralization brushing diminished mineral recovery, whereas exposure to mineral-rich beverage immediately after demineralization resulted in greater mineral deposition.

## Introduction

Dentin is a crucial component of teeth, consisting of approximately 70% inorganic material, 20% organic material, and 10% water [1, 2]. It provides structural support to enamel and facilitates sensory signal transmission within the tooth structure [3, 4]. The integrity of its inorganic component, primarily hydroxyapatite embedded in a collagen matrix, is essential for maintaining the tooth's structural resilience [5]. However, dentin is particularly vulnerable to acidic demineralization, which can severely compromise its functionality [6, 7].

Demineralization occurs when acids produced by bacterial metabolism or dietary sources dissolve the mineral content in dentin, weakening the tooth’s structure [8–10]. Conditions such as gingival recession, poor oral hygiene, and trauma lead to the exposure of dentin, further heightening its susceptibility to demineralization [11, 12]. Moreover, the use of highly abrasive or whitening toothpastes has been shown to exacerbate dentin erosion, thereby increasing the necessity for remineralization interventions [13–15]. Remineralization is the natural restorative process, wherein minerals—mostly calcium and phosphate—are redeposited onto demineralized dentin, restoring its integrity [16]. Saliva plays a vital role in this process, providing essential minerals and acting as a buffering agent to neutralize acidic conditions [17, 18]. However, the effectiveness of remineralization depends on environmental conditions, and natural remineralization can only restore mineral content to a limited extent [19].

Previous studies have explored the effects of intervention sequences, particularly the timing of brushing, on tooth surfaces, with most of the research focused on enamel [20–22]. Even within enamel studies, there are conflicting recommendations regarding the optimal timing of brushing. A recent literature review indicated that a larger number of studies found that brushing immediately after acidic exposure did not increase the risk of enamel wear when fluoridated toothpaste was used [23]. Some studies, however, suggested that delaying brushing by 30–60 min after acid exposure reduced wear on softened enamel and dentin, while a smaller number recommended individualized brushing times based on patient-specific needs [23–27]. This highlights the need for further research focused on dentin, along with the development of effective remineralization strategies to restore its structural integrity and prevent further degradation.

The objective of this study was to comprehensively evaluate the effects of demineralization, brushing, and remineralization on dentin, with a particular focus on the sequence of these interventions. X-ray fluorescence (XRF) spectroscopy was employed to analyze calcium and phosphorus content, profilometry to assess surface roughness, and Vickers hardness testing, along with scanning electron microscopy (SEM), to examine microhardness and structural changes. The study aimed to determine the most effective approach for preserving dentin’s mineral content and physical integrity. The findings are intended to guide clinical practices by identifying the optimal timing for brushing and remineralization, thereby minimizing dentin erosion and enhancing restorative outcomes.

## Materials and methods

### Specimen preparation and selection

**Fig. 1:**
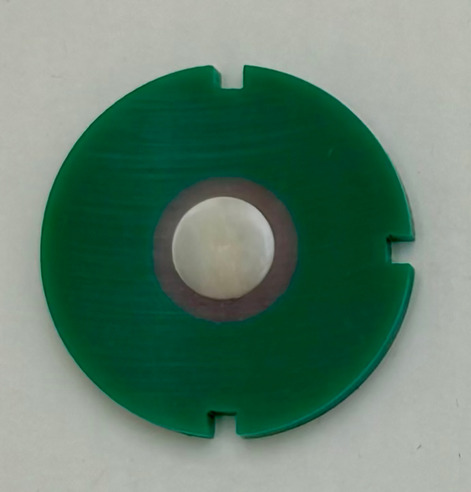
Bovine dentin specimen

Bovine dentin specimens (Fig. 1) were prepared using the methods employed in previous studies [14, 28]. Bovine teeth procured from the Korean Traditional Market in Seoul, Korea, and central and lateral incisors were extracted from the mandibular arch (no ethical approval was required according to the ethics committee of Seoul National University). Teeth exhibiting cracks or caries were discarded. Holes measuring 8 mm in diameter were drilled through the center of each tooth using a bench drilling machine (YDM-13 mm, Yongsoo Precision, Daegu, Korea) equipped with a cylindrical diamond core (⌀10 × ⌀8 mm) while supplying water to prevent overheating. Thereafter, the drilled teeth were affixed to custom-made acrylic rings (⌀30 × ⌀12 × 4 mm) using self-curing resin (ASCP3000500, Vertex-Dental, Soesterberg, Netherlands). The specimens were polished using a grinding and polishing machine (LaboPol-5, Struers, Copenhagen, Denmark) using silicon carbide papers (#220, #600, and #1200 SiC paper, R&B, Daejeon, Korea) to expose the dentin layer. Specimen thicknesses were measured after each grinding session using a digital micrometer (CD67-S15PM, Mitutoyo, Kawasaki, Japan) to ensure consistency in thickness.

The specimens for the experiment were selected based on predefined inclusion criteria—those having a surface roughness (Ra) of < 1 and a Vickers hardness number (VHN) of 50 ± 3 were considered satisfactory [29]. Using a contactless 3D surface profiler (NV-2400, NanoSystem, Daejeon, Korea), the Ra of dentin specimen was determined prior to the experiment. The Vickers hardness tester (HM-220, Mitutoyo, Tokyo, Japan) was used to measure the microhardness to ensure that each specimen consisted primarily of dentin. The sample size calculation was conducted using G*Power Version 3.1.9.6, based on a significance level of 0.05, a power of 0.95, and an effect size of 0.45. While the recommended total sample size was 100, the study opted to use 105 specimens to achieve an even distribution of specimens.

### Specimen treatment

**Fig. 2:**
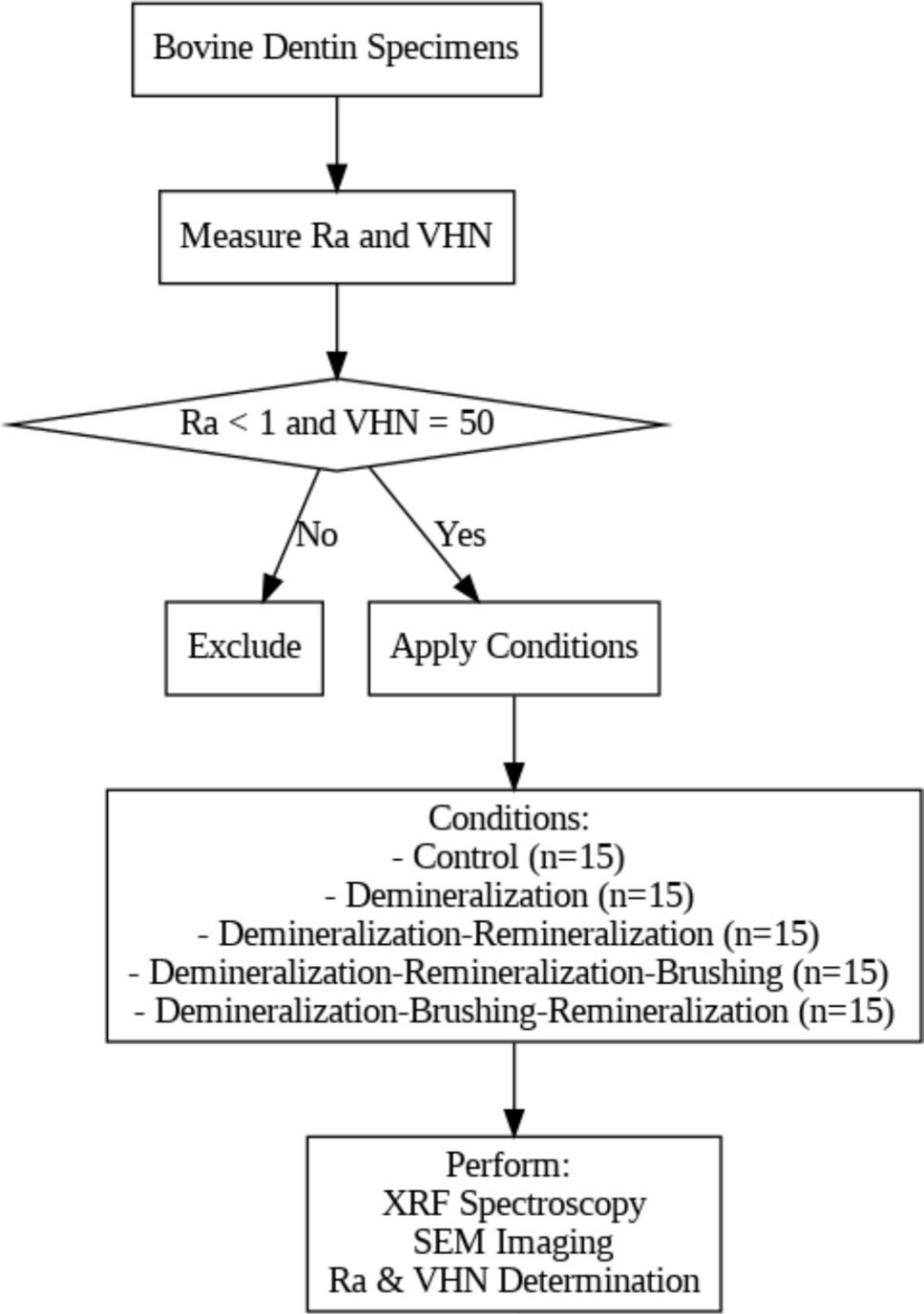
Flowchart illustrating the selection criteria and the applied conditions

For eligible tooth specimens, five different conditions were applied prior to the XRF analysis and SEM imaging (Fig. 2). Fifteen specimens were allotted for each condition:

A. Control (no treatment, C).
B. Demineralization (D).
C. Demineralization followed by remineralization (D–R).
D. Demineralization followed by remineralization, and then brushing (D–R–B).
E. Demineralization followed by brushing, and then remineralization (D–B–R).

The acid demineralization process involved immersing in dentin specimens in a citric acid buffer solution with a pH of 3 (Sigma-Aldrich, St. Louis, USA) for 20 min. The remineralization process involved immersion in whole milk (Namyang Dairy Products, Gangnam, Korea) for 20 min. Brushing was performed for 10 s using a brushing machine (RB118, R&B, Daejeon, Korea) and reference slurry. To prepare a reference slurry, a reference diluent solution containing 10% glycerin (99.5%, Shanghai Aladdin Biochemical Technology, Shanghai, China), 0.5% carboxymethyl cellulose (CMC, Sigma-Aldrich, St. Louis, USA), and 89.5% distilled water was created. The reference slurry was then prepared by mixing calcium pyrophosphate (99.95%, Strem Chemicals, Newburyport, USA) as a reference abrasive with the reference diluent solution in a 1:5 ratio per the ISO 11609 guidelines [30]. Specimens were rinsed with running water between treatments.

### X-ray fluorescence

An XRF analysis was conducted for the elemental analysis of dentin surface to assess the extent of remineralization and demineralization following treatments. The instrument was set to cover a wide measuring range from boron (B) to uranium (U), allowing for the detection of a broad spectrum of elements within dentin samples. The diameter of the analysis spot was standardized at 10 mm, providing a focused area for element detection and ensuring consistency across measurements. To minimize the interference from atmospheric elements and make the analysis more precise, a vacuum environment was maintained at a pressure of 5.5 Pa.

### SEM imaging

To visually verify the changes made on the dentin surface, a field emission scanning electron microscope (FE-SEM; Apreo S LoVac, ThermoFisher Science) was used. Specimens were coated with platinum prior to observation and energy-dispersive X-ray spectroscopy (EDS; XFlash 6160, Bruker) was used to determine the composition of various sections.

### Surface roughness and microhardness

To assess changes in surface roughness and microhardness after each treatment, 15 specimens were assigned to each of two experimental sequences. In the first group (sequence 1), the specimens underwent demineralization, followed by brushing, and then remineralization. In the second group (sequence 2), the specimens underwent demineralization, followed by remineralization, and then brushing.

### Statistical analysis

The one-way analysis of variance (ANOVA) was performed to determine if there were significant differences in the Ca/P among the specimens under different conditions. For the Ra and VHN data of the specimens subjected to serial treatments, a repeated-measures ANOVA was conducted to identify significant differences among the different treatment stages. Pairwise comparisons were subsequently performed using Tukey’s HSD test. All analyses were performed using Python 3.10.12.

## Results

**Fig. 3:**
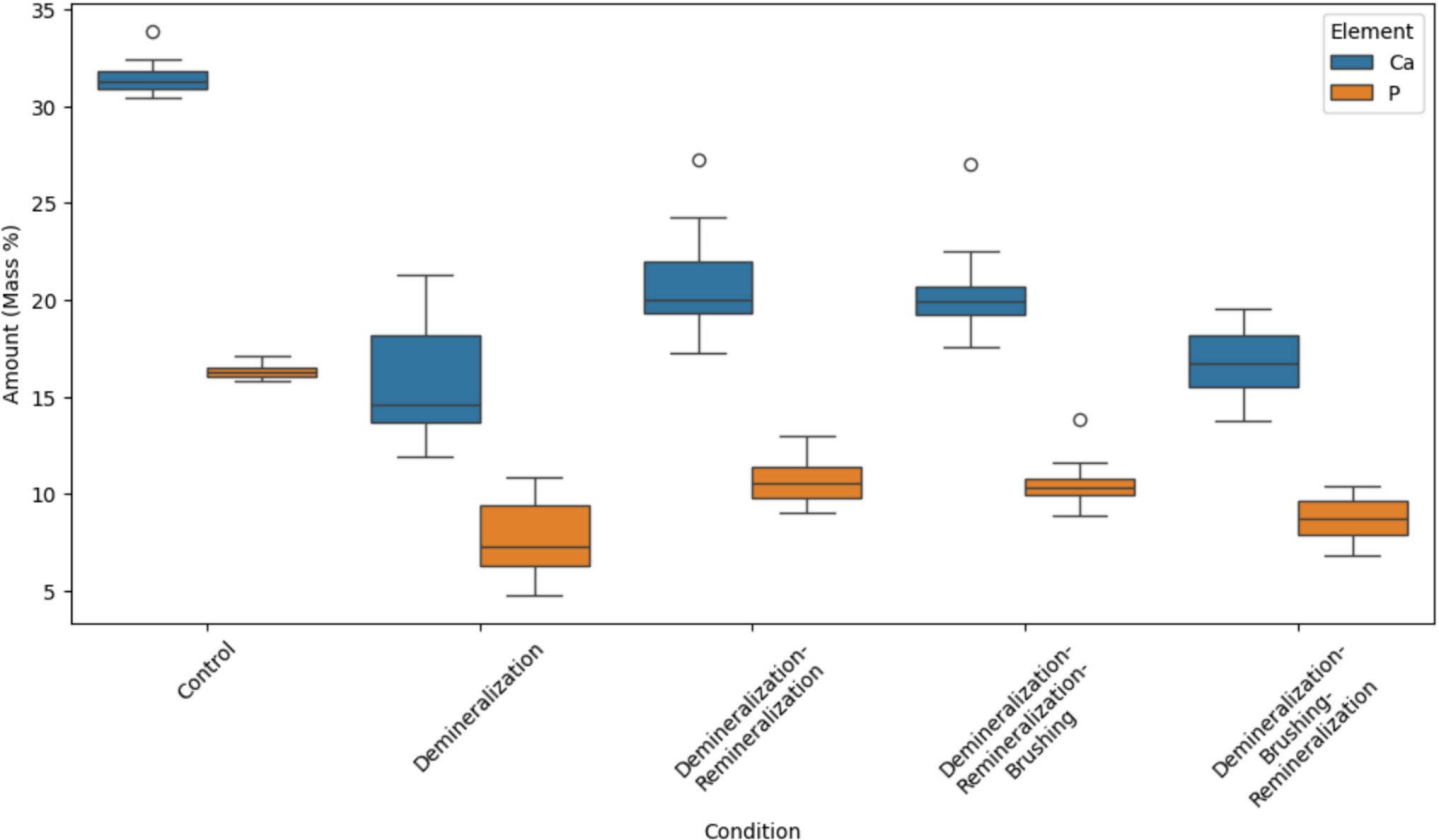
XRF analysis determining the amount of Ca/P in dentin specimens after applying each condition

As shown in Fig. 3, the Ca/P ratio was highest in the control (C) group, followed by the demineralization followed by remineralization (D–R) group, the demineralization followed by remineralization and brushing (D–R–B) group, the demineralization followed by brushing and remineralization (D–B–R) group, and lastly, the demineralization (D) group. Significant differences in calcium content were observed among the groups (*p* < 0.001), with pairwise comparisons revealing that the control group had significantly higher calcium content compared to the D–B–R (*p* < 0.001), D–R (*p* < 0.001), D–R–B (*p* < 0.001), and D (*p* < 0.001) groups (Table 1). The D–B–R group exhibited more calcium loss than the D–R group (*p* = 0.004), while the D–R–B group showed a significantly higher calcium content than the D–B–R group (*p* = 0.015). There was no significant difference between the D–B–R and D groups (*p* = 0.938), or between the D–R and D–R–B groups (*p* = 0.992). The phosphorus content followed a similar trend to calcium, with significant differences among most groups. The only non-significant differences were between the D–B–R and D groups, as well as between the D–R and D–R–B groups (Table 1).

**Table 1:** Comparisons of the Ca/P content based on the treatment administered

C vs. D–B–R	< 0.001^**^	< 0.001^**^
C vs. D–R	< 0.001^**^	< 0.001^**^
C vs. D–R–B	< 0.001^**^	< 0.001^**^
C vs. D	< 0.001^**^	< 0.001^**^
D–B–R vs. D–R	0.004^*^	0.022^*^
D–B–R vs. D–R–B	0.015^*^	0.043^*^
D–B–R vs. D	0.938	0.469
D–R vs. D–R–B	0.992	0.999
D–R vs. D	< 0.001^**^	< 0.001^**^
D–R–B vs. D	0.002^*^	< 0.001^**^

^*^p < 0.05

^**^p < 0.001

**Fig. 4:**
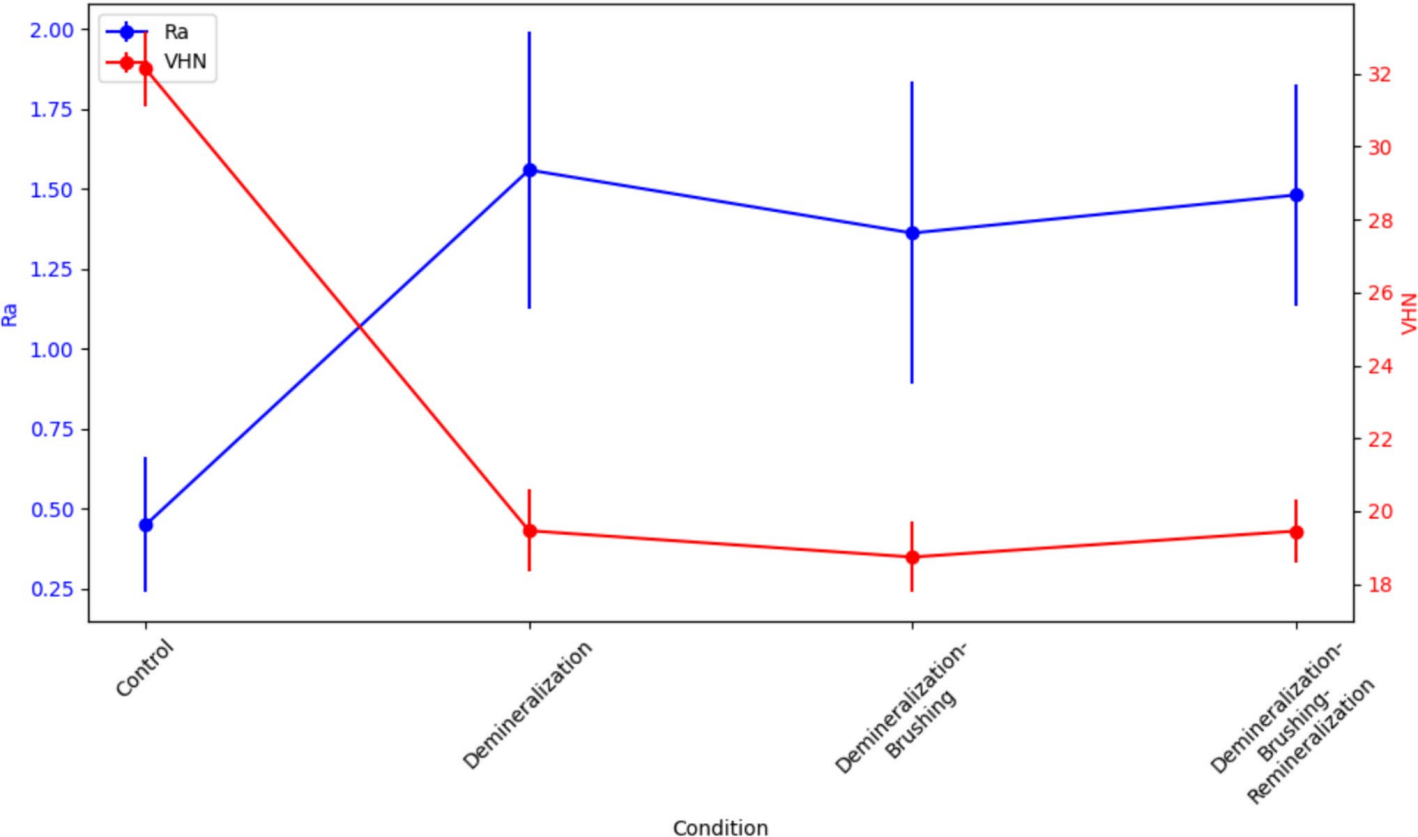
Surface roughness and microhardness measurements for sequence 1

**Fig. 5:**
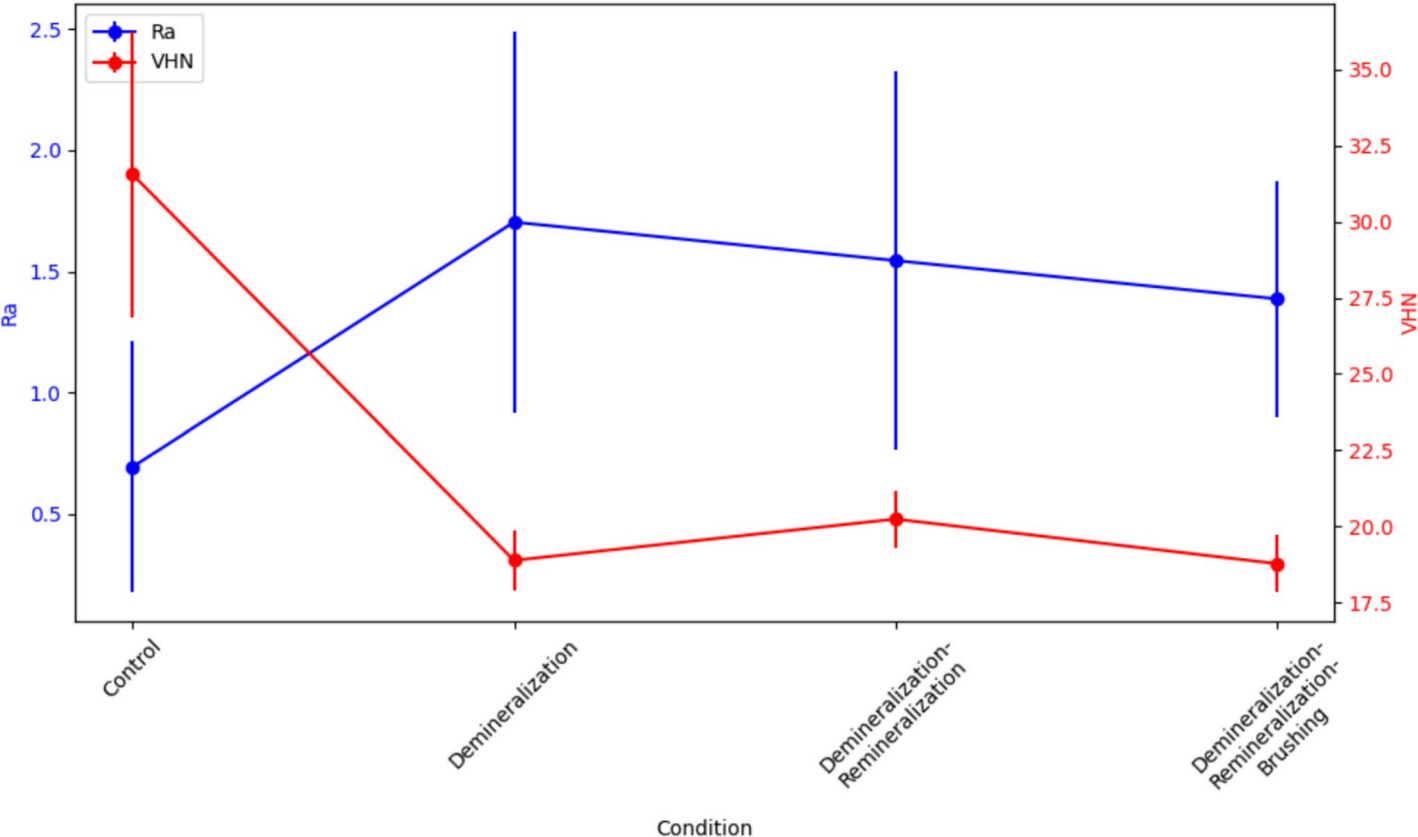
Surface roughness and microhardness measurements for sequence 2

A repeated-measures ANOVA showed significant differences in both Ra and VHN across the treatment conditions for both experimental sequences (*p* < 0.001) (Tables 2 and 3, Figs. 4 and 5). In the first sequence (D–B–R), Ra increased significantly after each treatment step (*p* < 0.001), although there were no significant differences between the D–B and D–B–R groups (*p* = 0.882). VHN showed a significant decrease after each treatment step (*p* < 0.001), but no significant differences were found between the D–B and D–B–R groups (*p* = 0.353) (Table 2).

**Table 2:** Repeated-measures comparisons of surface roughness and microhardness for sequence 1

Pairs	*p* value (Ra)	*p* value (VHN)
C vs. D	< 0.001^**^	< 0.001^**^
C vs. D–B	< 0.001^**^	< 0.001^**^
C vs. D–B–R	< 0.001^**^	< 0.001^**^
D–B vs. D–B–R	0.882	0.353
D vs. D–B	0.617	0.341
D vs. D–B–R	0.962	1.0

^*^p < 0.05

^**^p < 0.001

**Table 3:** Repeated-measures comparisons of surface roughness and microhardness for sequence 2

Pairs	*p* value (Ra)	*p* value (VHN)
C vs. D	0.0001^**^	< 0.001^**^
C vs. D–R	0.008^*^	< 0.001^**^
C vs. D–R–B	0.014^*^	< 0.001^**^
D vs. D–R	0.335	0.164
D vs. D–R–B	0.242	0.997
D–R vs. D–R–B	0.997	0.110

^*^p < 0.05

^**^p < 0.001

In the second sequence (D–R–B), Ra and VHN also differed significantly across treatment conditions (*p* < 0.001). Significant increases in Ra were observed in all treatment groups compared to the control, but no significant differences were found between the D–R and D–R–B groups (*p* = 0.997). Similarly, microhardness decreased significantly in all treatment groups compared to the control, with no significant differences between the D–R and D–R–B groups (*p* = 0.110) (Table 3).

**Fig. 6:**
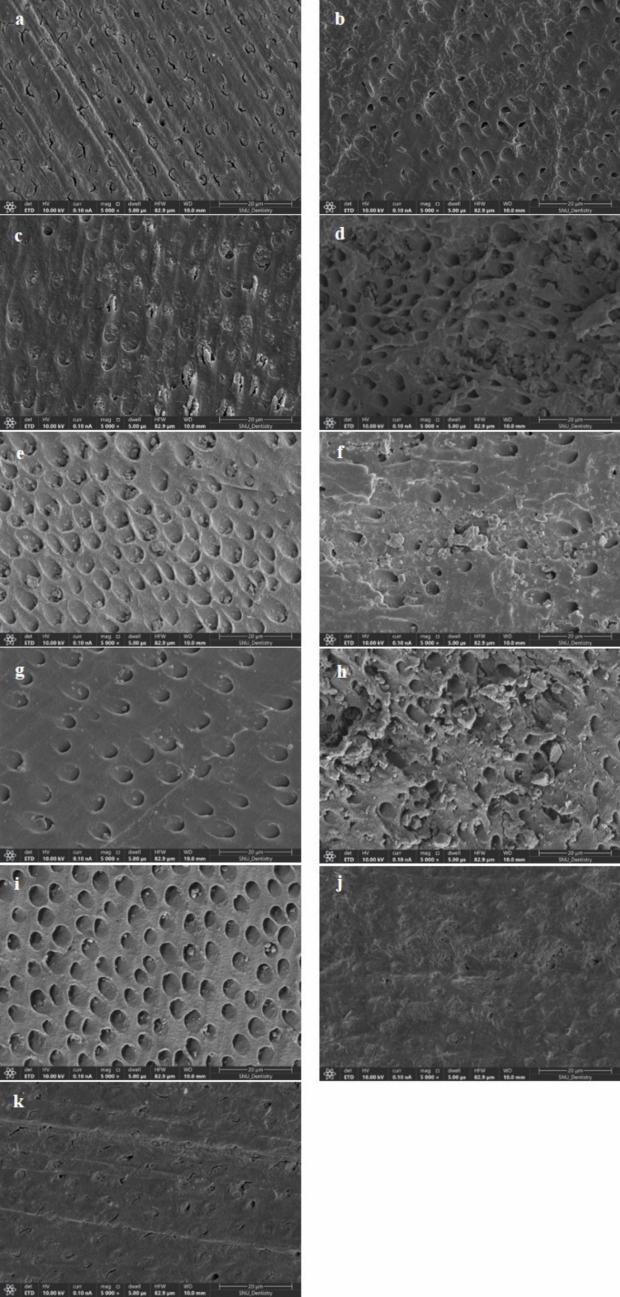
SEM images of dentin surface following the different treatments: **a** control (no treatment), **b** D, **c** D–R, **d** D–R–B (brushed region), **e** D–R–B (unbrushed region), **f** D–B (brushed region), **g** D–B (unbrushed region), **h** D–B–R (brushed region), **i** D–B–R (unbrushed region), **j** brushed-only (brushed region), **k** brushed-only (unbrushed region)

**Fig. 7:**
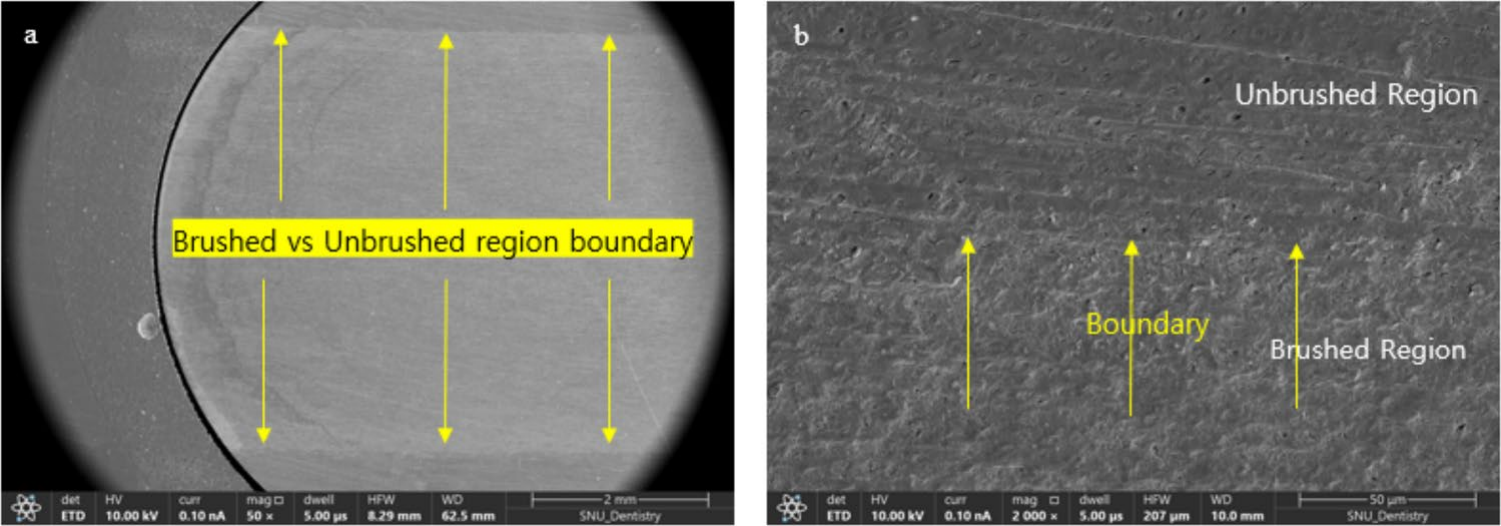
**a** SEM image showing the brushed and unbrushed region boundaries. **b** SEM image showing distinct differences between brushed and unbrushed regions with visible brush marks

SEM images confirmed the treatment-dependent effects, with visible dentinal tubule exposure in all groups except the control. Brushed regions showed significantly more damage, with clear distinctions between brushed and unbrushed areas (Figs. 6 and 7).

## Discussion

XRF spectroscopy has emerged as an effective method for analyzing the elemental composition of dental tissues, providing both high sensitivity and precision in detecting mineral changes [31, 32]. In this study, XRF was employed to assess changes in dentin composition following acidic demineralization, tooth brushing, and the application of a weak remineralizing substance, such as milk, to restore minerals on eroded dentin surfaces [33, 34]. The extent of hydroxyapatite loss can be measured by quantifying the levels of calcium and phosphorus ions, as XRF effectively determines the retention of these elements. Hydroxyapatite, or Ca_10_(PO_4_)_6_(OH)_2_, consists of 3.38% hydroxyl groups, 18.5% phosphorus, and 39% calcium by weight, allowing calcium and phosphorus as key indicators of its structural integrity [35, 36]. When exposed to acidic conditions, hydroxyapatite releases calcium and phosphate ions, which buffer the environment and mitigate further demineralization [37, 38]. However, with prolonged acid exposure, the release of these ions becomes insufficient, eventually leading to the breakdown of hydroxyapatite, as demonstrated by significant calcium ion release under bacterial acid exposure [37].

The present study’s results revealed that the timing and sequence of treatments had a significant impact on the Ca/P content of dentin. The control group, which received no treatment, retained a significantly higher Ca/P content compared with the other treatment groups. These findings indicate that the demineralization process, followed by brushing, remineralization, or both, leads to a significant loss of hydroxyapatite compared with the untreated group.

Among the treated groups, significant differences in Ca/P retention were observed. Specifically, the D–R group had a significantly higher Ca/P content compared with the D–B–R group, suggesting that brushing after demineralization and before exposure to milk results in greater hydroxyapatite loss, whereas directly proceeding to remineralization after demineralization is more effective in mineral content preservation.

Interestingly, no significant difference in Ca/P content was observed between the D–B–R group and the D-alone group, indicating that exposure to milk after brushing does not significantly alter the Ca/P content. Additionally, no significant difference was found between the D–R group and the D–R–B group, suggesting that remineralization should be performed immediately after demineralization to effectively restore Ca/P levels. The significant differences observed between the D group and both the D–R group and the D–R–B group underscore the beneficial effect of remineralization immediately after demineralization in mitigating acid-induced Ca/P loss.

Surface roughness and microhardness did not return to their baseline levels, similar to the Ca/P content, though the effects of remineralization were less pronounced on roughness and microhardness compared with Ca/P levels. After demineralization, the subsequent treatment sequences did not show significant differences, indicating that the remineralization was insufficient and brushing did not significantly impact dentin’s surface roughness or hardness. However, microhardness results indicated that brushing after acid treatment or remineralization negatively affected dentin’s microhardness. There was a trend toward decreased VHN following brushing, though this decrease was not statistically significant. This suggests that while some mineral deposition occurred, it did not significantly influence surface roughness or microhardness, possibly due to the complexity of the remineralization process.

In general, it is the enamel, and not dentin, that is primarily exposed to the oral environment. The former is also susceptible to acidic demineralization and undergoes remineralization [39]. However, the global trend of population aging has led to an increased frequency of dentin exposure in the oral environment due to natural aging-related changes, as well as pathological changes such as gingival recession, attrition, and non-carious cervical lesions [40]. Additionally, dentin hypersensitivity, which frequently occurs when dentin is exposed but the exposure is not limited to it, is a frequently encountered presenting complaint in dental clinics [41]. Dentin, with its higher organic content compared with enamel, may exhibit different patterns of acidic demineralization and remineralization [42].

Based on the experimental results of this study, brushing immediately after demineralization tended to decrease the VHN slightly, along with a reduction in Ra values. In contrast, when a remineralization phase followed demineralization, the Ra value decreased to a similar extent; however, there was a slight increase in VHN though not statistically significant. The changes in surface roughness due to remineralization differed between the two sequences. Specifically, in sequence 1, where brushing preceded remineralization, the Ra value increased during the remineralization phase, while in sequence 2, where remineralization preceded brushing, the Ra value decreased. Conversely, the VHN values increased slightly in both sequences.

Several points can be considered based on these results. The amount of remineralization was insufficient compared with demineralization, and the same 20-min demineralization and remineralization periods used in this study may not have been sufficient for remineralization considering the relatively weaker potential of milk as a remineralizing agent. Conversely, the use of citric acid with a pH of 3 was intended to simulate conditions similar to carbonated drinks, juice, or coffee, which are commonly consumed by many people; however, the 20 min of application may represent a more severe environment than the oral cavity, which has a natural buffering system, though the evident surface change was intended for experimental purposes.

Some hypotheses can be proposed as to why better results were obtained when remineralization preceded brushing. First, the surface of demineralized dentin may retain an organic matrix, which can serve as a useful precipitation bed for remineralization. Therefore, if this physically weakened layer is brushed off, the efficiency of remineralization may decrease. Figures 3 and 4 suggest that during the remineralization phase, regardless of the sequence of events, the Ra values as well as VHN showed slight increments, which may indicate that the remineralization process does not inherently restore the original smooth surface and that the process was not fully mature. After sufficient remineralization, it might be beneficial to incorporate a polishing process, similar to the final step in restorative treatment, to reduce surface roughness for better oral hygiene, and brushing could potentially serve this purpose, at least to some extent. Nevertheless, further research is needed to thoroughly investigate the differences in dimensions and patterns between brushing-induced roughness and demineralization-induced roughness.

The findings also suggest that smoother surfaces may facilitate mineral deposition more effectively than rougher ones do, perhaps because of the relationship between surface uniformity and remineralization. The rougher surface created by brushing may hinder stable mineral deposition, whereas a more uniform surface provides a better environment for mineral deposition. A previous study by Tschoppe, et al. [43] revealed that smaller hydroxyapatite particles (20 nm) adhere better and form a more uniform layer on the enamel’s surface, which remains stable even under ultrasonic treatment. This stability and uniformity suggest that surfaces that were rendered smoother by the application of smaller particles are more effective for mineral deposition and enamel repair. This possibly explains why the Ca/P content was higher in conditions where brushing was either excluded or performed after mineral deposition, suggesting that minerals are better able to accumulate on smooth or relatively less damaged surfaces.

Although these results were obtained from in vitro experiments, previous studies have demonstrated the limited remineralizing capacity of artificial saliva, while the remineralizing effects of milk have been somewhat confirmed [44–46]. From the perspective of the general public, even rinsing the mouth with milk, which is rich in calcium and phosphorus, after consuming an acidic drink could be beneficial. This practice could potentially aid in dentinal remineralization, a process crucial for managing hydraulic conductance, which is key to understanding and treating dentin hypersensitivity.

Hydraulic conductance measures the ease with which fluids flow through dentinal tubules, with higher values indicating increased fluid flow that can trigger nerve endings in the dental pulp, causing pain and sensitivity [47, 48]. A study by Kijsamanmith, et al. [49] demonstrated the effectiveness of various types of milk in reducing hydraulic conductance by occluding dentinal tubules. In our study, remineralization treatments post-demineralization influenced dentin’s calcium content, suggesting that effective occlusion of dentinal tubules and reduction of hydraulic conductance could enhance the efficacy of remineralization processes. This underscores the importance of selecting appropriate agents and treatment sequences that not only restore mineral content but also mitigate fluid flow within dentinal tubules to alleviate sensitivity.

The effectiveness of remineralization in restoring dentin’s structural integrity depends not only on the presence of mineral ions but also on the method of their delivery. Bertassoni, et al. [50] demonstrated that continuous remineralization, where minerals are consistently supplied to the D dentin, significantly enhances the recovery of mechanical properties, with up to 60% of normal dentin’s strength restored. This approach involves continuously replenishing the remineralizing solution with Ca^2+^ and PO_4_^2−^, maintaining a stable environment that promotes deep mineral integration within the dentin matrix. In contrast, static remineralization methods, in which the solution is not replenished and mineral deposition occurs until the solution reaches equilibrium, were shown to yield only superficial mineral deposits and minimal mechanical recovery. These findings align with the observations of this study whereby mineral deposition alone did not significantly restore the microhardness of dentin specimens. Therefore, for functional recovery of dentin, the continuous delivery of remineralizing agents is more crucial than mere surface mineral deposition.

The findings have significant implications for clinical practice, particularly in the development of effective remineralization therapies and preventive measures against dentin hypersensitivity and caries progression. The findings of this study suggest that one-time applications of remineralizing agents may suffice for achieving long-term dentin restoration. Instead, sustained or repeated application protocols could be necessary to ensure deeper and more stable mineral integration, leading to better preservation of dentin’s mechanical properties. Moreover, the impact of brushing on reducing the effectiveness of remineralization highlights the need for careful consideration of oral hygiene practices immediately following remineralization treatments. Clinicians may need to advise patients on avoiding abrasive toothpaste or delaying brushing after consuming acidic drinks to minimize tooth demineralization.

## Conclusion

This study demonstrates that the sequence of demineralization, brushing, and remineralization treatments significantly affects the retention of calcium and phosphorus in dentin, as well as its surface roughness and hardness. Demineralization led to substantial hydroxyapatite loss, which was only partially restored by subsequent remineralization. Notably, brushing before remineralization further reduced mineral recovery, highlighting the importance of the order of therapeutic interventions. While definitive conclusions cannot be drawn from this single in vitro study, it may be advisable to recommend brushing after consuming a beverage containing calcium and phosphorus rather than immediately after consuming an acidic beverage. These findings have important implications for clinical practice, suggesting that sustained remineralization strategies, careful post-acidic food consumption hygiene practices, and the incorporation of Ca/P containing products could improve dentin restoration outcomes.

## Data Availability

The data that support the findings of this study are available under reasonable request.
